# Complete Genome Sequence of *Flagellimonas* sp. Strain CMM7, Isolated from a Marine Green Alga, *Codium minus* (Schmidt) Silva

**DOI:** 10.1128/mra.00153-22

**Published:** 2022-05-16

**Authors:** Jang-In Shin, Man-Seok Bang, Ji Young Kim, Chung-Hun Oh

**Affiliations:** a Department of Oral Physiology, College of Dentistry, Dankook University, Cheonan, Choongnam, Republic of Korea; b Department of Medical Laser, Graduate School, Dankook University, Cheonan, Choongnam, Republic of Korea; c Research Institute for Basic Science, Jeju National University, Jeju City, Jehu, Republic of Korea; Montana State University

## Abstract

Here, we report the genome sequence of *Flagellimonas* sp. strain CMM7, which was isolated from a marine green alga, *Codium minus* (Schmidt) Silva, in Jeju Island, South Korea. The genome is complete and consists of 4,421,981 bp, with a GC content of 37.5%, 3,942 predicted protein-coding sequences, and 49 RNA genes.

## ANNOUNCEMENT

The genus *Flagellimonas* is a member of the family *Flavobacteriaceae* of the phylum *Bacteroidetes* (1). *Flagellimonas* sp. have been reported to have been isolated from various marine algae, including Ecklonia kurome (2), Ecklonia cava (3), Asparagopsis taxiformisand (4), and Hymeniacidon sinapium (5).

Twenty-five species of seaweed were collected from the subtidal zone in Biyang-do, Jeju-do, South Korea (33°41′996″N, 126°22′549″E). The collected samples were sufficiently washed with sterile seawater to remove foreign substances adhering to the surface of the seaweed. After the addition of 1 g of filtered sterile seawater to the washed seaweed, it was ground using a homogenizer. Each sample was suspended in 0.85% (wt/vol) saline, serially diluted, and plated on marine agar (MA) (Difco) plates, followed by incubation aerobically at 25°C for 2 weeks. Microorganisms with different colony shapes were subcultured on new MA plates at 25°C for 4 days. The isolated microorganisms were stored in 20% glycerol and stored frozen at −80°C.

The genomic DNA of the isolated microorganisms was extracted using the Wizard genomic DNA purification kit (Promega, USA) according to the protocol recommended by the manufacturer. The quantity and quality of the isolated DNA were determined using a NanoDrop spectrophotometer. Sequencing of the 16 rRNA gene was performed using the 27F/907R and 785F/1492R primers with the isolated microorganism, and it was confirmed that the microorganism isolated from a marine green alga, Codium minus (Schmidt) Silva, was *Flagellimonas* sp. strain CMM7.

To sequence the whole genome of *Flagellimonas* sp. strain CMM7, a SMRTbell library with a 15- to 20-kb insert size using the BluePippin size selection system was constructed with the Pacific Biosciences (PacBio) DNA template preparation kit v1.0. The genome was sequenced with the PacBio RS II sequencing platform, using a single-molecule real-time (SMRT) Cell 8Pac v3 and DNA/polymerase binding kit P6 reagents, by Macrogen (Seoul, South Korea) (6). In total, 119,041 PacBio subreads (average subread length, 7,020 bp; subread *N*_50_, 10,564 bp) of *Flagellimonas* sp. strain CMM7 were generated.

*De novo* assembly was performed using the FALCON-integrate protocol v2.1.4 (7). Default parameters were used except where otherwise noted. When the ends of the contigs overlapped, the contigs were connected to form a circular DNA. The result of the assembly was one contig, which consisted of one closed circular chromosome of 4,421,981 bp (GC content, 37.5%; coverage, 148×). The genomes were annotated with NCBI Prokaryotic Genome Annotation Pipeline (PGAP) v5.3 (8), which identified 3,991 genes, 3,942 coding DNA sequences (CDSs), 6 rRNAs, 39 tRNAs, and 4 noncoding RNAs on the chromosome.

### Data availability.

The complete genome sequence of *Flagellimonas* sp. strain CMM7 was deposited in GenBank under the accession number CP090003. The associated BioProject, BioSample, Assembly, and SRA accession numbers are PRJNA789932, SAMN24175386, ASM2139019v1, and SRX13462110, respectively.
